# Brf1-Mediated RNA Polymerase III Activity Limits Murine Gammaherpesvirus Spread

**DOI:** 10.64898/2026.09.14.751424

**Published:** 2026-09-17

**Authors:** K Rapchak, LS Nyberg, LA Johnson, RA Elbert, CJ Huff, ZA Grissom, SE Dremel, JM Tucker

## Abstract

RNA polymerase III (Pol III) activity is upregulated during herpesvirus infection, yet the functional consequences of this response remain poorly understood. To investigate the role of host Pol III transcription during murine gammaherpesvirus 68 (MHV68) infection, we depleted the Pol III transcription factor Brf1, an essential component of the TFIIIB complex required for transcription from Type I and Type II Pol III promoters. Brf1 depletion enhanced MHV68 replication during low multiplicity of infection (MOI) conditions, resulting in increased viral gene expression, viral protein accumulation, infectious virion production, and extracellular viral genome copies. These effects were confirmed to be Brf1-specific through rescue with an siRNA-resistant Brf1 construct. In contrast, Brf1 depletion produced only modest effects during high-MOI infection, suggesting that Brf1-dependent antiviral activity is most important during multistep viral spread. Transcriptomic analysis revealed accelerated induction of interferon-responsive genes early during infection in Brf1-deficient cells, followed by enhanced host transcript depletion at later stages, consistent with amplified host shutoff. Genetic disruption of the RIG-I/MAVS signaling pathway failed to abolish Brf1 antiviral activity, indicating that this phenotype is independent of MAVS-dependent interferon signaling. Finally, plaque assays demonstrated that Brf1-deficient cells supported larger plaques and increased plaque numbers, suggesting enhanced viral spread and/or entry. Together, our results reveal an unexpected role for Brf1-dependent Pol III activity in controlling gammaherpesvirus spread and suggest that virus-induced Pol III activation contributes to host antiviral defense.

**Importance:**

RNA polymerase III (Pol III) activity is stimulated by DNA tumor virus infection, yet the biological significance of this response has remained unclear. Utilizing the murine gammaherpesvirus, MHV68, we demonstrate that the Pol III transcription factor Brf1 functions as a previously unrecognized antiviral restriction factor. Depletion of Brf1 enhanced viral gene expression, protein accumulation, infectious virion production, plaque formation, and viral spread. Genetic rescue was able to restore restriction of infection. Surprisingly, this phenotype was most pronounced during low-multiplicity infections and was associated with accelerated viral cell-cell spread. Transcriptomic analysis revealed that Brf1 influences host antiviral responses and the dynamics of virus-induced host shutoff, highlighting an unexpected connection between Pol III transcription and cellular defense pathways. Importantly, Brf1-mediated restriction occurred independently of the canonical RIG-I/MAVS signaling axis. These findings provide new insight into why Pol III transcription is broadly induced during herpesvirus infection and identify Brf1-dependent Pol III activity as a key host mechanism that limits gammaherpesvirus spread.

## Full Text Availability

The license terms selected by the author(s) for this preprint version do not permit archiving in PMC. The full text is available from the preprint server.

